# Task‐Specific Modulation of Cognitive Control: Electrophysiological Evidence From Bivalency Effect in Task Switching

**DOI:** 10.1002/pchj.70011

**Published:** 2025-03-26

**Authors:** Yunfei Cao, Jianxiao Wu, Gege Liu, Fen Sun, Fuhong Li

**Affiliations:** ^1^ School of Psychology Jiangxi Normal University Nanchang China; ^2^ School of Teacher Education Chengdu University Chengdu China; ^3^ School of Business Administration Nanchang Institute of Technology Nanchang China; ^4^ Department of Student Affairs Heilongjiang Polytechnic Harbin China

**Keywords:** bivalency effect, bivalent stimuli, conflict inhibition, ERP, task switching

## Abstract

An occasional presence of bivalent stimuli in a block of univalent trials can elicit a slowing of the response on all subsequent univalent trials. This type of modulation of cognitive control is termed the bivalency effect. To explore whether this modulation is task specific, this study used a triplet task switching paradigm, with three following tasks that were presented concussively: a shape color judgment (red vs. blue), a number parity judgment (odd vs. even), and a letter case judgment (lowercase vs. uppercase). The event‐related potential (ERP) results showed that (1) the bivalency effect was reflected by the decreased amplitude of N2 and P3a over the frontal region for both the color and letter tasks; (2) the bivalency effect occurred earlier for the color task compared with that for the letter task; (3) for the number parity task, the bivalency effect was observed in the increased N1 and the decreased P2p over the parietal region. These findings indicate that the modulation of cognitive control is task‐specific after the presentation of bivalent stimuli in task switching.

## 1 Introduction

Task switching is one of the typical paradigms in the study of cognitive control (Cao et al. 2023; Chen et al. 2022; Gratton et al. 2018; Schuch et al. 2019; Vandierendonck et al. 2010; Yancey et al. 2022; Dann et al. 2023). When faced with complex tasks, individuals need to continuously adjust their cognitive control to adapt to changing contexts and task requirements. In task‐switching studies, a variety of experimental stimuli types have been employed to examine the underlying processing mechanisms associated with switching costs. Experimental stimuli are typically classified as either univalent or bivalent based on their dimensional attributes. Univalent stimuli are characterized by having a singular task‐relevant feature (e.g., color, font size), while bivalent stimuli are those that encompass features from two or more tasks (Grundy et al. 2013; Woodward et al. 2003, 2008; Dann et al. 2023). The most frequently used stimuli in task‐switching studies are bivalent stimuli (Vandierendonck et al. 2010). Research has shown that in task switching, bivalent stimuli not only have a longer response time than univalent stimuli, exhibiting a greater switching cost (Allport and Wylie 2000), but also affect the processing of subsequent univalent stimuli (Enger 2009; Woodward et al. 2003). This effect is referred to as the bivalency effect (Du and Li 2018; Grundy et al. 2013); studies of the cognitive neural mechanisms underlying the bivalency effect can help to further understand the brain mechanisms underlying cognitive control and its modulation processes.

A triplet task with a fixed sequence is frequently used in studying the bivalency effect, including a shape color judgment (red vs. blue), a number parity judgment (odd vs. even), and a letter case judgment (lowercase vs. uppercase). In most cases, the letter is colored in black. Occasionally, the letter appears in red color, which cues features from both the color task and the letter task; for example, when encountering the red letter B, participants are required to ignore the color to make a letter case judgment (Grundy and Shedden 2014a; Strobach et al. 2018; Woodward et al. 2003). The bivalency effect refers to a blockwise slowing of responses to all the subsequent univalent stimuli after an occasional bivalent stimulus (Botvinick et al. 2001; Moretti et al. 2024; Grundy et al. 2019; Meier et al. 2009; Metzak et al. 2013; Rey‐Mermet and Meier 2012a; Rey‐Mermet et al. 2013; Rey‐Mermet and Meier 2014, 2014b, 2017; Woodward et al. 2003, 2008).

There are two main theories explaining the bivalency effect, which are the episodic context binding account and the history‐dependent predictive model. According to the episodic context binding account, a cognitive conflict caused by bivalent stimuli is associated with the context. The reactivation of the complex representation of this episodic context interferes with the performance in all subsequent univalent trials. In the face of conflict, the performance of the cognitive control system is decelerated, resulting in the bivalency effect (Meier et al. 2013; Rey‐Mermet and Meier 2014b; Moretti et al. 2023). A study by Meier et al. (2013) verified the episodic context binding theory. They compared the performance of amnesiacs with that of normal participants. The results showed that the normal group showed a sustained slowing of response time in all three tasks in mixed blocks (containing both univalent and bivalent stimuli) compared with that in univalent blocks. By contrast, the amnesic patients only showed slowed responses in the first task after the bivalent stimulus, with a shorter duration of the bivalency effect. Researchers suggest that the task‐context bundle is disrupted due to memory deficits in amnesic patients (Allport and Wylie 2000; Meier et al. 2009).

The history‐based predictive model indicates that the bivalency effect reflects the adjustment of cognitive control mainly in suppressing the impact of irrelevant features of bivalent stimuli on the current and subsequent tasks (Grundy and Shedden 2014a; Sheth et al. 2012). Compared with congruent bivalent stimuli (e.g., the same response key for two stimulus properties), incongruent bivalent stimuli generate a larger bivalency effect. The event‐related potential (ERP) results demonstrated that the activity of the dorsal anterior cingulate cortex (dACC) was greater for trials following incongruent bivalent trials than for trials following congruent bivalent trials, indicating an increased response of the dACC to the high levels of conflict produced by incongruent bivalent stimuli (Grundy and Shedden 2014b). Accordingly, the bivalency effect reflects a process involving the dACC, by which future cognitive load can be predicted on the basis of the current and recent cognitive demands (Grundy and Shedden 2014a, 2014b; Sheth et al. 2012).

Both the episodic context binding account and the history‐dependent predictive model emphasize the modulating role of cognitive control in conflict behaviors, which is associated with the dACC (Ebitz et al. 2020; Sheth et al. 2012; Smith et al. 2019; Giordano et al. 2023). Neuroimaging studies demonstrated that the activation of the dACC and the pre‐supplementary motor regions is associated with the bivalency effect (Woodward et al. 2008). ERP studies have shown that the early ERP components related to the bivalency effect appear in the temporo‐parietal junction, reflecting the early visual process. Moreover, compared with those in univalent blocks, univalent stimuli in bivalent blocks evoked more positive amplitudes in the 300–600 ms time window (Grundy et al. 2013), which were in the same polarity as the difference between the bivalent and univalent stimuli (Poulsen et al. 2005).

The bivalency effect has been demonstrated to be unaffected by a number of factors, including stimulus characteristics (Meier et al. 2009), presentation (Meier et al. 2009), response keystrokes (Rey‐Mermet and Meier 2012a), and age (Rey‐Mermet and Meier 2014). These findings support the stability and generalizability of the bivalency effect. In contrast, some studies have indicated that the magnitude of the bivalency effect is influenced by conflict properties (Rey‐Mermet and Meier 2012a) and practice (Grundy et al. 2013). For example, univalent stimuli that had relevant features to the bivalent stimuli had a similar bivalency effect in the switching and repetition conditions. However, univalent stimuli without relevant features to the bivalent stimuli showed a bivalency effect only in the switching condition, while the bivalency effect was reduced in the repetition condition (Rey‐Mermet and Meier 2012a). The results of these studies indicated that the bivalency effect might not be task general but task specific (Grundy et al. 2013).

Although some ERP studies have attempted to reveal the neural mechanism of the bivalency effect, they have only compared the electrophysiological differences evoked by all univalent stimuli in univalent and bivalent blocks (Grundy et al. 2013, 2019; Grundy and Shedden 2014a), but have not distinguished the bivalency effect of different task types (e.g., color, case, and parity). Therefore, two behavioral studies have indicated that the bivalency effect may be task‐specific (Grundy et al. 2013; Rey‐Mermet and Meier 2012a). However, the neural mechanisms underlying the task‐specific bivalency effect remain unclear. Indeed, in the bivalent blocks, the cognitive conflict arises from the bivalent stimulus in the letter case task and might trigger the cognitive control of inhibiting (or at least ignoring) the conflict or interference. The conflict information is associated with the shape color task and the letter case task, but not with the number parity task. In light of the above, we hypothesize that the modulation of conflict by cognitive control is more likely to be a task‐specific rather than a task‐general process (Liu et al. 2013; Egner et al. 2007; Junker et al. 2023), which is possibly reflected by the observation that the bivalency effect of different task types may differ in different brain regions and in different time windows (e.g., ERP components).

To verify this hypothesis, a triplet task with a fixed sequence (Grundy and Shedden 2014a; Strobach et al. 2018; Woodward et al. 2003) and the ERP technology with high‐temporal‐resolution measurements were adopted in this study. Given that each task is associated with a distinct relationship with the bivalent stimulus, our hypothesis was that all three tasks would demonstrate the bivalency effect on reaction time (RT). However, the ERP results may reveal the task‐specific nature of the bivalency effect. Specifically, when a bivalent stimulus is presented, the interference (or conflict) monitoring module of cognitive control (Botvinick et al. 2001; Yancey et al. 2022) transmits the detected interference information to the conflict resolution module, which is required to inhibit the irrelevant information and select the correct behavior. Thus, the increased cognitive control constantly modulates the response to subsequent trials. The modulation by cognitive control might be task‐specific. That is, following the bivalent stimuli (a colored letter), the subsequent color task requires overcoming or ignoring the carryover of the inhibition of color, the subsequent number parity task is performed with the enhanced attentional alertness (Posner and Petersen 1990), and the subsequent letter task is primarily a process of conflict (or interference) adaptation. All these differences in the modulation of cognitive control might be reflected by the observation of the bivalency effect, which may have different spatial distributions (electrode) and temporal properties of the ERP components in different task types.

## 2 Methods

### 2.1 Participants

Thirty‐five university students (22 females), ranging in age from 18 to 24 years, with an average age of 21.6 years, participated in the experiment. All participants were selected from the broader community at Jiangxi Normal University, and it should be noted that none of them were pursuing a degree in psychology. They were right‐handed, had normal or corrected‐to‐normal vision, were not color blind, and had no history of mental illness. Before the experiment, G*Power 3.1 software was used to estimate the sample size. The effect size was set to 0.25 (medium size, using Cohen's *d*), the power level was 0.95, the *α* level was 0.05, and the required sample size was 26. The study was approved by the ethics committee of the School of Psychology at Jiangxi Normal University and complied with the latest version of the Declaration of Helsinki. All participants provided written informed consent.

### 2.2 Materials and Design

**FIGURE 1:**
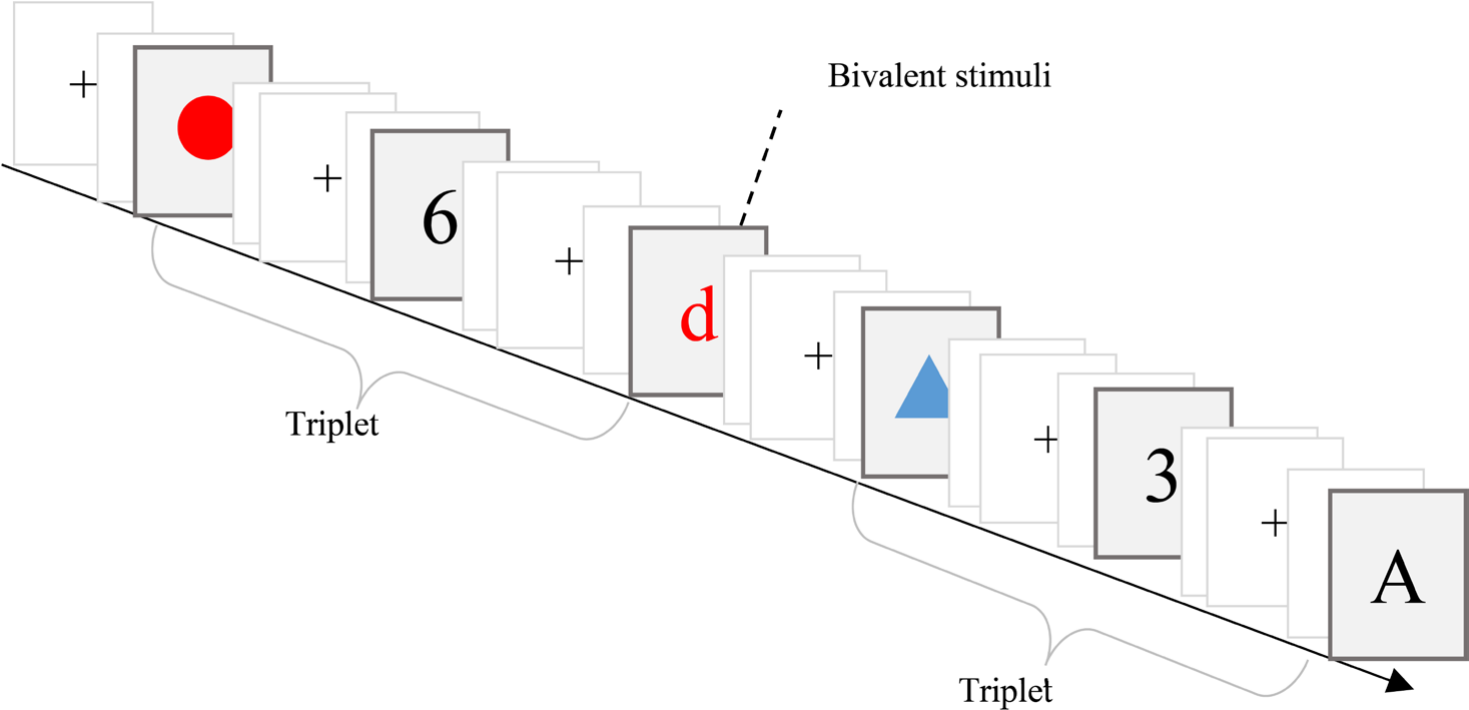
Illustration of the trial sequence and type of stimuli used during the experiment.

A triplet task with a fixed sequence is the classic paradigm for exploring the bivalency effect in task switching literature (Grundy et al. 2013; Grundy and Shedden 2014a; Woodward et al. 2003). Accordingly, the following three tasks were used with a fixed sequence: a shape color judgment, a number parity judgment, and a letter case judgment. For the univalent stimuli, the shape color task required classifying shapes as red or blue, the letter case task required classifying letters (a, b, e, f) as upper or lower case, and the number parity task required classifying numbers (1–8) as odd or even. Bivalent stimuli were letters with a color, such as red or blue; that is, a letter might appear in blue or red, cueing both the relevant letter case judgment and the irrelevant color judgment. The participants were required to press the “F” key for red shapes, odd digits, or uppercase letters and the “J” key for blue shapes, even numbers, or lowercase letters. The correspondence between key presses and stimulus properties was balanced across participants. Bivalent stimuli were only presented in the bivalent blocks, and participants were told to ignore the color of the letters merely to make the letter case judgment (Figure 1).

The entire experiment was divided into practice and experimental sessions. The practice session was presented with 20 univalent stimuli for the three tasks. Eight experimental blocks consisted of two univalent and six bivalent blocks. Each block contained 180 trials, where blocks 1 and 8 were univalent blocks, and blocks 2–7 were bivalent blocks. The univalent blocks included 60 shape color trials, 60 number parity trials, and 60 letter case trials. The shape color and number parity trials were the same in the bivalent block and the univalent block; the only difference was that the letters in the bivalent block were occasionally presented with red (or blue) letters as bivalent stimuli. Bivalent stimuli were presented pseudo‐randomly, with 12 bivalent stimuli included in each bivalent block, resulting in a total of 72 trials. At the beginning of each trial, a fixation cross was presented at the center of the screen for 500 ms, followed by a random blank screen with a randomized duration of 500–800 ms. Stimuli remained visible on the screen until the participant responded or for 1000 ms, and a blank screen was presented for 500–800 ms after each stimulus.

### 2.3 Data Collection and Analysis

E‐Prime 2.0 was used to present the stimuli and to collect behavioral data, and all trials of each condition were used to examine accuracy. Meanwhile, erroneous responses and RT outliers (defined as ±2.5 SD from the average RT of each condition) were excluded from the analysis of RT. The proportion of eliminated trials of the color task, number parity task, and letter task in the univalent block and the bivalent block was approximately 5.4%, 7.8%, 5.9%, 8.3%, 6.7%, and 7.5%, respectively.

An electroencephalogram (EEG) was recorded using 64 Ag/AgCl electrodes in accordance with the international 10–20 system. The bandpass filter was set to 0.01–100 Hz, and the sampling frequency was 500 Hz. The online reference electrode was positioned on the FCz, while the ground electrode was positioned on the AFz. The vertical electrogram electrode was positioned below the right eye. All inter‐electrode impedances were maintained at a level below 10 kΩ. Subsequently, the ERPs were analyzed offline using Brain Vision Analyzer 2.1.2 (Brain Products, Munich), with re‐referencing to the two mastoid channels (TP9 and TP10). Independent component analyses were conducted to exclude trials that exhibited eye movements, blinks, motion, or other artefacts in any channels. Subsequently, the ERPs were subjected to digital filtering in order to remove frequencies below 0.1 Hz or above 30 Hz (30 dB). Trials exhibiting electrooculography (EOG) artefacts (defined as an absolute difference between two sampling points exceeding 50 μV/ms, peak‐to‐peak deflections of more than 100 μV, or amplitudes exceeding ±80 μV for a segment over a 100 ms interval) and trials contaminated by artefacts were excluded from the averaging process. The extracted epochs (from −100 to 500 ms) of the correct trials were time‐locked to the target onset and were baseline‐corrected (−100 to 0 ms). Following the rejection of artefacts, a minimum of 40 clean trials was obtained for each condition and participant. The artefact‐free EEGs for each participant in each condition were averaged.

A multivariate statistical approach, partial least squares (PLS; Lobaugh et al. 2001; McIntosh and Lobaugh 2004), was employed, which does not impose any prior assumptions regarding the temporal or spatial characteristics of the effects. This method enabled the refinement of temporal intervals and spatial regions of interest for subsequent conventional statistical analyses. Previous studies have found that the brain region associated with the bivalency effect is primarily located in the frontal cortex, while the parieto‐occipital region plays a critical role in the recognition of visual stimulus features (Grundy et al. 2013; Woodward et al. 2008). Consequently, the analysis focuses on electrophysiological variations recorded from electrodes positioned over the frontal and parieto‐occipital regions. Repeated‐measures analysis of variance (ANOVA) and paired‐samples t‐tests were conducted at specific time points and spatial locations of interest. The PLS analysis was employed to explore the bivalency effect. Bootstrap analysis of electrode salience, offering confidence intervals for salience across time points and electrodes, revealed that in the shape‐color task, the effect was most robust in frontal electrodes (FPz, FP1, FP2, AF3, AF4, AF7, AF8, Fz, F1, F2, F3, F4, F5, F6, F7, F8) during time windows of 145–190 ms, 275–315 ms, and 335–400 ms. Furthermore, significant effects were observed in parieto‐occipital electrodes (Pz, P1, P2, P3, P4, P8, PO4, PO7, PO8) within 160–175 ms and 275–450 ms. In the number parity task, the bivalency effect was most reliable in frontal electrodes (FPz, FP1, FP2, AF3, AF4, AF7, AF8, Fz, F1, F2, F3, F4, F5, F6, F7, F8) during time windows of 155–220 ms, 270–320 ms, and 425–500 ms. Additionally, parieto‐occipital electrodes (Pz, P1, P2, P3, P4, P8, PO4, PO7, PO8) showed significant effects from 155–200 ms, 230–250 ms, and 270–415 ms. For the letter case task, the effect was most pronounced in frontal electrodes (FPz, AF3, AF4, Fz, F1, F2, F3, F4, F5, F6, F7) between 360 and 455 ms. Moreover, parieto‐occipital electrodes (P7, PO7) exhibited significant effects from 175–220 ms.

To further examine these findings, traditional componential statistical tests were applied to the bivalency effect in the frontal region and, for comparative purposes, in the parieto‐occipital region. Components were selected through visual inspection and their agreement with the PLS findings. Therefore, 10 electrode sites (F1, F2, F3, F4, F5, F6, AF3, AF4, AF7, and AF8) over the frontal region (Grundy et al. 2013; Poulsen et al. 2005) and six electrode sites (P7, P8, PO3, PO4, PO7, and PO8) over the parieto‐occipital region (Poulsen et al. 2005) were selected for analysis. The electrode sites were combined to form four areas of interest, which were the left frontal region (F1, F3, F5, AF3, and AF7), right frontal region (F2, F4, F6, AF4, and AF8), left parieto‐occipital region (P7, PO3, and PO7), and right parieto‐occipital region (P8, PO4, and PO8). The preliminary analyses showed that the time window of the visible bivalency effect exhibited notable differences between tasks. Hence, the ERPs were analyzed separately in each task. The ERP components were selected based on visual inspection and correspondence with the results of previous relevant studies (Grundy et al. 2013; Rey‐Mermet et al. 2013). In the shape color task, we analyzed the mean amplitudes of P2 (175–225 ms), N2 (275–325 ms), P3a (350–400 ms), and LPC (400–500 ms) over the frontal region and those of N1 (155–185 ms) and P3b (250–440 ms) over the parieto‐occipital region by performing a 2 hemisphere (left or right) × 2 block type (univalent or bivalent) repeated‐measures ANOVA. In the number parity task, we analyzed the mean amplitudes of N1 (150–200 ms) and P2p (200–280 ms) of the parieto‐occipital region by performing a 2 hemisphere (left or right) × 2 block type (univalent or bivalent) repeated‐measures ANOVA. In the letter case task, we also analyzed the mean amplitudes of specific ERP components. Previous studies have been unable to directly compare the ERP components of bivalent stimuli with those of univalent stimuli due to the small number of bivalent stimulus trials (Du and Li 2018; Grundy et al. 2013; Grundy and Shedden 2014a, 2014b). However, in the present study, 72 trials were set up to be bivalent stimuli, which allowed for a sufficient number of superimposed averages to be obtained during EEG preprocessing. Furthermore, bivalent stimuli were included in the statistical analyses. Therefore, there were three types of letter stimuli: letters in univalent blocks, univalent letters in bivalent blocks, and bivalent letters in bivalent blocks. We analyzed the mean amplitudes of P2 (185–235 ms), N2 (280–330 ms), and P3a (450–500 ms) over the frontal region by performing a 2 hemisphere (left or right) × 3 letter type (letter in univalent block, univalent letter in bivalent block, bivalent letter) repeated‐measures ANOVA. The Greenhouse–Geisser correction and Bonferroni adjustments were applied to adjust ANOVA and *p* values of multiple comparisons.

## 3 Results

### 3.1 Behavioral Results

The mean RTs for each task in different blocks are presented in Table 1. We compared the RT of the relevant univalent stimuli in all the univalent blocks and bivalent blocks (2–7). A 2 (block type: univalent, bivalent) × 3 (task type: shape color, number parity, letter case) repeated‐measures ANOVA was performed on the RTs of univalent stimuli in these blocks. The results showed that there was a main effect of task type, *F*(2,68) = 49.91, *p* < 0.001, *η* ^2^ = 0.59, indicating that participants were faster in responding to stimuli in the shape color task and the letter case task compared to the number parity task. Specifically, multiple comparisons revealed significant differences in reaction times between the number parity task and the other two tasks (*p*s < 0.001), while no significant difference was found between the shape color task and the letter case task (*p* > 0.1). The main effect of block type was non‐significant (*p* = 0.499). To investigate whether the non‐significant effect of block type (i.e., bivalency effect) was due to a practice effect, we additionally analyzed the first bivalent block, following the methodology of Grundy et al. (2013). The results of this analysis are presented in the Appendix.

**TABLE 1:** Mean RTs (ms) for univalent stimuli in the univalent and bivalent blocks.

	Univalent block	Bivalent block (2–7)
Shape color task	513 (52)	516 (58)
Number parity task	565 (58)	561 (56)
Letter case task	526 (54)	520 (48)

### 3.2 ERP Results

**FIGURE 2:**
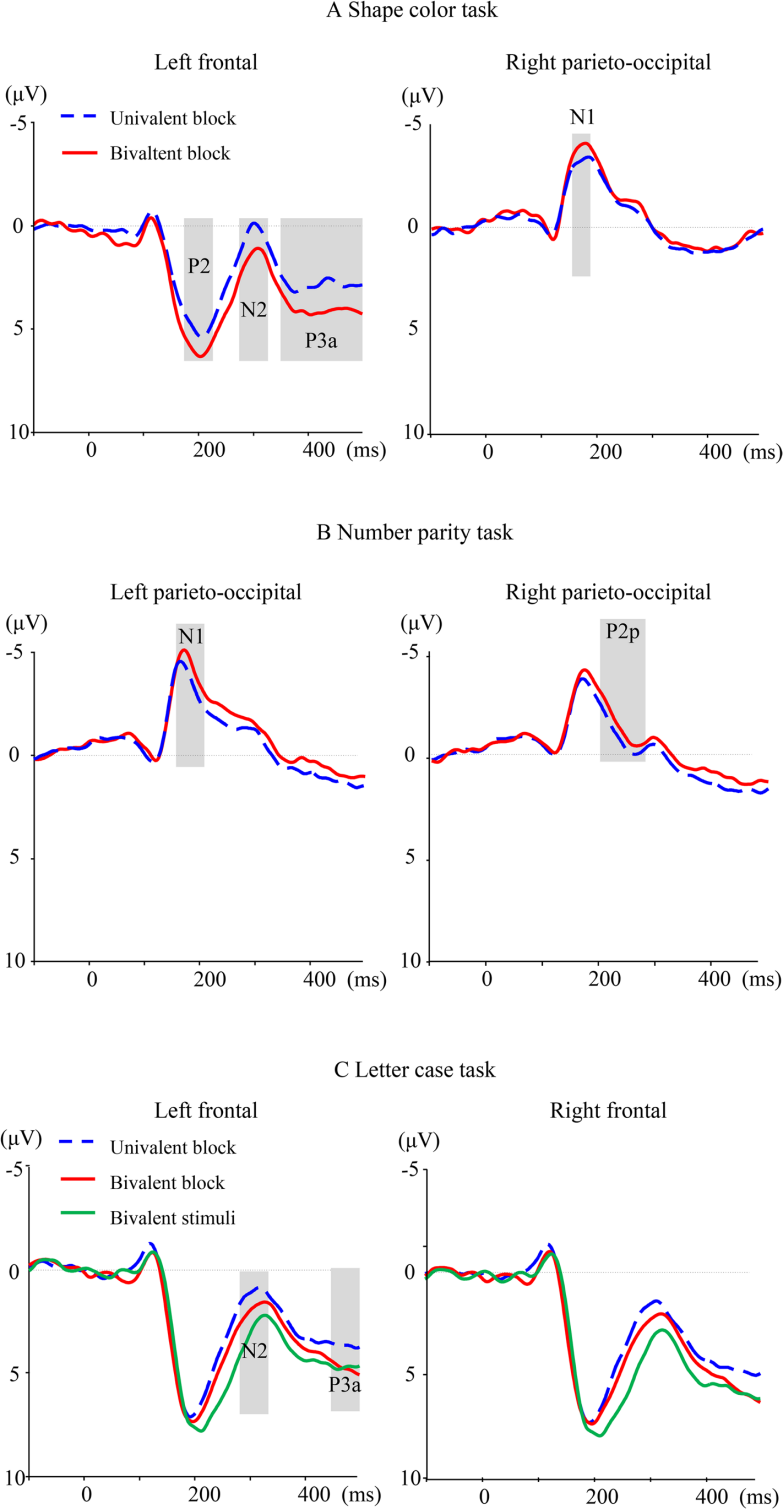
ERP waveforms of the bivalency effect across three tasks.

The grand averaged EEG waveforms of univalent stimuli of the three tasks in different block types are shown in Figure 2. The EEG data analysis related to the bivalency effect of different tasks is as follows.

#### 3.2.1 The Shape Color Task

The bivalency effect was observed in four time windows (Table 2), 175–225 ms (P2), 275–325 ms (N2), 350–400 ms (P3a), and 400–500 ms (LPC), over the frontal region. Over the parietal region, the bivalency effect was only found in the 155–185 ms (N1) time window. The ANOVA of the EEG data over the frontal region showed that in the 175–225 ms (P2) time window, the main effect of block type was significant, *F*(1, 34) = 4.58, *p* = 0.039, *η* ^2^ = 0.11, with larger P2 amplitudes in the bivalent block than in the univalent block. In the 275–325 ms (N2) time window, the main effect of block type was significant, *F*(1, 34) = 6.18, *p* = 0.018, *η* ^2^ = 0.15, with smaller N2 amplitudes in the bivalent block than in the univalent block. In the 350–400 ms (P3a) time window, the main effect of block type was significant, *F*(1, 34) = 5.76, *p* = 0.022, *η* ^2^ = 0.14, with larger P3a amplitudes in the bivalent block than in the univalent block. In the 400–500 ms (LPC) time window, the main effect of block type was significant, *F*(1, 34) = 13.70, *p* = 0.001, *η* ^2^ = 0.28, with larger LPC amplitudes in the bivalent block than in the univalent block. The ANOVA over the parieto‐occipital region showed that in the 155–185 ms (N1) time window, the main effect of block type was significant, *F*(1, 34) = 4.46, *p* = 0.042, *η* ^2^ = 0.11, with larger N1 amplitudes in the bivalent block than in the univalent block.

**TABLE 2:** Brain region and time windows (ms) that the bivalency effect presented in among different tasks.

	Frontal region	Parietal region
Shape color task	175–225, 275–325, 350–400, 400–500	155–185
Number parity task	—	150–200, 200–280, 380–440
Letter case task	280–330, 450–500	—

#### 3.2.2 The Number Parity Task

No bivalency effect was found in any time window over the frontal region. However, over the parieto‐occipital region, the bivalency effect was observed in the following two time windows: 150–200 ms (N1) and 200–280 ms (P2p). The result of ANOVA on the amplitudes of the 150–200 ms (N1) time window showed the main effect of block type, *F*(1, 34) = 4.57, *p* = 0.040, *η* ^2^ = 0.11, with larger N1 amplitudes in the bivalent block than in the univalent block. In the 200–280 ms (P2p) time window, the main effect of block type was significant, *F*(1, 34) = 7.15, *p* = 0.011, *η* ^2^ = 0.17, with smaller P2p amplitudes in the bivalent block than in the univalent block.

#### 3.2.3 The Letter Case Task

No significant bivalency effect was found in any time window over the parietal region. The result of ANOVA on the mean amplitudes of 185–235 ms (P2) over the frontal region showed that there was no significant difference in the amplitudes evoked by univalent and bivalent letters in the bivalent block (*p* = 0.176). The results of ANOVA on the amplitude in 280–330 ms (N2) indicated that the main effect of letter type was significant, *F*(2, 68) = 12.01, *p* < 0.001, *η* ^2^ = 0.26. Multiple comparisons showed that the N2 amplitudes evoked by bivalent letters were smaller than those evoked by univalent letters in the bivalent block (*p* = 0.034) and in the univalent block (*p* < 0.001). The N2 amplitudes evoked by univalent letters in the bivalent block were smaller than those in the univalent block (*p* = 0.047). In the 450–500 ms (P3a) time window, the main effect of letter type was significant, *F*(2, 68) = 3.62, *p* = 0.036, *η* ^2^ = 0.09. Multiple comparisons showed that the P3a amplitudes evoked by bivalent letters were larger than those evoked by univalent letters in the univalent block (*p* = 0.037), and the amplitudes of univalent letters in the bivalent block were larger than those in the univalent block (*p* = 0.022).

## 4 Discussion

### 4.1 Behavioral Results and Their Implications

Behavioral results demonstrated no significant bivalency effect when we analyzed all the six bivalent blocks, which is inconsistent with the results of previous studies (Du and Li 2018; Meier et al. 2009; Metzak et al. 2013; Rey‐Mermet and Meier 2012a, 2012b). However, the bivalency effect appeared pronounced in the first three bivalent blocks (see Appendix), which is consistent with the findings of Grundy et al. (2013). In their study, a practice effect was observed across all three tasks, indicating that the bivalency effect decreases or even disappears with extended practice. Both the study of Grundy et al. (2013) and the present study have compared the task‐specific magnitude of the bivalency effect, but there is a discrepancy in behavioral results between these two studies. Grundy et al. (2013) found that the magnitude of the bivalency effect was greater for the color task than for the other two types of tasks, whereas there is no significant difference in our studies. The most likely reason for this discrepancy is that the number of bivalent blocks (i.e., 6) is greater in our study than that of Grundy et al. (2013) which comprises three bivalent blocks in total.

### 4.2 ERP Results and Cognitive Control Modulation

Most importantly, the ERP results with high temporal precision showed that the bivalency effect appeared differently in the different types of task, demonstrating that the modulation of cognitive control is task‐specific. Firstly, the spatial (electrode sites) distribution of the bivalency effect is task‐specific. For both the shape color task and the letter case task, the bivalency effect appeared in the anterior region, but for the number parity task, the bivalency effect did not appear in this region. The most possible explanation is that the number parity task had no overlapping features with the bivalent stimuli; thus, there was minimal conflict information in completing this task, and no bivalency effect was found in the prefrontal region that is associated with conflict processing. During task switching, the conflict information originating from features of the bivalent stimuli needed to be monitored in the dACC (Woodward et al. 2008). In the present study, two features of bivalent stimuli were color and letter. We found that the shape color task and the letter case task, which are related to the two features of bivalent stimuli, not the number parity task, evoked the frontal N270 component, reflecting the need for the modulation of cognitive control (Enger 2009; Larson et al. 2014; Mao and Wang 2011; Syrov et al. 2022).

Because the perceptual properties of bivalent stimuli (i.e., letters with color) did not appear in the number parity task, there was neither a process of deinhibition nor a process of conflict adaptation after the disappearance of interference in the processing of the number parity task. Therefore, no modulation of cognitive control in the prefrontal lobes, particularly the ACC, was involved. The bivalency effect for the number parity task was only observed in the parietal region, which might be related to the enhancement of early attention and alertness in digit processing. According to the attention network model proposed by Posner and Rothbart (2007), the activation of the parietal cortex is related to the allocation of attention resources. In bivalent blocks, the presence of bivalent stimuli, although indirectly linked to the processing of number parity, may enhance cognitive control and, in turn, increase attentional alertness for the number parity task. This increased alertness and the associated cognitive control modulation share similarities with the concept of mixed costs (Mari‐Beffa and Kirkham 2014) and the influence of task context on cognitive control (Cai et al. 2023; Zhuo et al. 2021).

Furthermore, the time course of the bivalency effect is task‐specific. The ERP results demonstrated that both the shape‐color and number‐parity tasks elicited a more negative N1 component over the parietal sites in the bivalent blocks compared with the univalent blocks. This finding is consistent with previous studies (Grundy et al. 2013), which suggest that the additional early visual‐perceptual processing needed during bivalent blocks may be reflected in the N1 component. A number of relevant ERP studies have demonstrated that the early attention‐related component, such as P1/N1, is an effective indicator of deinhibition (Chen et al. 2022; Giller and Beste 2019; Wolff et al. 2018). This reflects the important role of the inhibition gated mechanism and attention selection mechanism in the deinhibition process (Chen et al. 2022; Giller et al. 2020; Koch et al. 2004). In the present study, the color judgments share a feature (color) with the bivalent stimuli, a feature that is irrelevant and must be ignored or suppressed on bivalent trials. The color task is the initial task following the presentation of a bivalent stimulus, which requires the disinhibition of the color feature and the allocation of attention toward the color task. It can thus be posited that the negative N1 component triggered by the color task in this study may reflect the crucial role played by the inhibition gated mechanism and attention selection mechanism in the deinhibition process (Chen et al. 2022; Giller et al. 2020; Koch et al. 2004). Similarly, the number parity task evoked the N1 component over the parietal sites. However, this component reflected attentional enhancement rather than disinhibition, and the number parity task was not associated with the bivalent stimulus. In contrast, within the mixed blocks, the enhancement of cognitive control prompted by the presence of bivalent stimuli may enhance attentional alertness in the number parity task.

In the N2 time window, the bivalency effect occurred in both the color and letter tasks but not in the number parity task; that is, the amplitude of N2 evoked by these two tasks was decreased in the bivalent blocks compared with that in the univalent blocks. This effect may be indicative of an adjustment in cognitive control processes triggered by the presentation of a bivalent stimulus. The N2 component peaks at 280–340 ms, is distributed over the frontocentral area, and is associated with conflict detection (Gajewski et al. 2018; Jost et al. 2017; Scannella et al. 2016; Wang et al. 2001, 2003) and inhibition control during response selection (Clayson and Larson 2013; Gajewski et al. 2018). The present study employed a fixed‐sequence triplet task paradigm, in which the participants were not required to repeat the same task but rather to switch between different tasks. N2 is sensitive to task switching (Gajewski et al. 2018; Han et al. 2018; Nicholson et al. 2006; Swainson et al. 2003; Xie et al. 2020). During task switching, variations in N2 wave amplitude may have reflected the monitoring of inter‐task conflict and the allocation of cognitive control resources. Specifically, while performing the current task, participants were required to monitor whether the current task was the same as the previous task while inhibiting the previous task. In short, the previous tasks must be suppressed while the new tasks are being performed. In the bivalent blocks, however, the inclusion of two perceptual features in the bivalent stimulus required participants to allocate cognitive control resources for the inhibition of information unrelated to the target tasks. This may have resulted in a reduction in the cognitive control resources available for inhibiting the old tasks, which in turn would be reflected in the decreased N2 amplitude. Therefore, the decreased N2 amplitude observed in bivalent blocks in the present study may not directly reflect the increased conflict (or interference) from the bivalent stimuli themselves but rather the decreased cognitive control required to inhibit the previous tasks in task switching resulting from those conflicting interferences. It is important to note that the modulation of cognitive control in response to N2 amplitude was only manifested in the color task and the letter task and did not appear in the parity task, suggesting that the modulation of cognitive control triggered by the bivalent stimulus is task‐specific.

The process of regulation of cognitive control also appeared in P3a. Bivalent stimuli evoked larger positive waves than univalent stimuli during the time window of 300–600 ms (Poulsen et al. 2005), and the univalent stimuli in the bivalent blocks evoked larger positive waves than the univalent stimuli in the univalent blocks (Grundy et al. 2013). The similarities in the ERP components may suggest an overlap in processes related to the bivalency effect and the conflict generated by the bivalent stimuli. As mentioned above, the bivalent stimuli triggered the modulation of cognitive control, which may persist in the univalent stimuli. In our study, there were two tasks associated with bivalent stimuli: one was the color task, and the other was the letter task. The color task in the bivalent blocks evoked more positive waves in both the 350–400 ms and 400–480 ms time windows than that in the univalent blocks, which may reflect the increased task‐set reconfiguration process after the inhibition was released. In other words, the color feature was irrelevant information in the bivalent stimuli and needed to be inhibited to perform the letter task correctly. However, when performing the subsequent color task, participants needed to overcome the inhibition of the color feature and reconfigure it into the task‐set. The letter task in the bivalent blocks also evoked more positive waves in the P3a (300–440 ms) time window than that in the univalent blocks. Further comparison of the univalent and bivalent stimuli in the letter task showed that bivalent letter stimuli evoked more positive amplitudes than univalent letter stimuli in the 340–390 ms time window, indicating that there was a process of conflict resolution in the letter task (Grundy et al. 2013; Poulsen et al. 2005).

### 4.3 Theoretical Implications of Task‐Specific Bivalency Effects

The task‐specific nature of the bivalency effect revealed by the ERP results in this study supported the view of a history‐dependent predictive model, not an episodic context binding account. According to the episodic context binding account, binding contains not only stimuli, responses, and tasks but also the context related to such stimuli, responses, and tasks. The RT slowing observed as the bivalency effect was the result of the cautious response style of participants while dealing with the complex context created by bivalent stimuli, which might have affected the processing of subsequent univalent stimuli within the block (Meier et al. 2009). According to this account, the three tasks were bound together as a whole, so there should be no task specificity, and the ERP components related to the bivalency effect of the three tasks should be identical. However, this study showed that the bivalency effect of the three tasks evoked different ERP deflection over different scalp regions, so the episodic context binding account cannot well explain the task specificity of the bivalency effect in ERP data. By contrast, the history‐dependent predictive model suggested that the dACC encodes the conflict generated by bivalent stimuli, which raises the cognitive load of prediction for upcoming trials. This prediction leads to response slowing when subsequent univalent trials appear. The present study examined the processing of conflict (conflict monitoring as well as conflict control) by manipulating the different relationships between univalent and bivalent stimulus features. The results supported the hypothesis that the dACC is specialized in continuously updating the predicted demand for cognitive resources. This account was particularly sensitive to relative changes in the complexity of different contexts and is weighted by the recent past. The significant influence of current dACC activity on future neural activity and behavior allowed the implementation of behavioral adjustments to optimize performance (Clayson and Larson 2013; Sheth et al. 2012). In the mixed blocks, the color task following the bivalent stimulus required the individual to release the inhibition of the color feature and then to respond to the color task with a keystroke, reflecting the process of de‐inhibition; the subsequent parity task, although not associated with the bivalent stimulus, reflected an increase in attentional alertness as the individual remained alert to the task due to the presence of the bivalent stimulus; and the subsequent letter task was free of the color, in comparison to the bivalent stimulus feature interference, reflecting the process of attentional regrouping in a distraction‐free situation. The ERP results of this experiment demonstrated the different spatial distribution and temporal properties of the bivalency effect evoked by univalent stimuli with different relationships to the bivalent stimuli in the mixed blocks, which further supported that this experience‐based modulation of cognitive control is task‐specific.

### 4.4 Limitations of the Study

The present study sought to explore the task‐specific aspects of cognitive control through a fixed‐triplet task paradigm. Nevertheless, the following limitations must be acknowledged. Firstly, it must be acknowledged that the present study is merely exploratory in nature, and thus the reliability of the conclusions drawn must be further confirmed. For instance, it would be beneficial to ascertain whether the temporal window of the ERP components associated with the task‐specific aspects of the bivalency effect is stable across experimental contexts and also needs to be further verified. Secondly, the present study employed a triplet task with a fixed sequence to investigate the task‐specific aspects of the bivalency effect. The bivalent stimuli were always presented only in the letter task. This means that the task identity and the position from the bivalent trial might be confounded. Despite previous studies having found that the sequence position effect was found not to affect the bivalency effect for each task, suggesting that the order of the tasks plays little role in generating the bivalency effect (Rey‐Mermet and Meier 2012a). In future studies, it would be beneficial to utilize a non‐fixed triple task in order to eliminate the potential influence of sequence position.

## 5 Conclusion

This study explored the task specificity of the bivalency effect through ERP technology. The ERP results, (1) the bivalency effect was observed in the N2 and P3 components in the frontal region for both the color and letter tasks, possibly reflecting the process of conflict adaptation; (2) compared with the letter‐case task, the shape‐color task had an earlier and longer time window of the bivalency effect, which may reflect different processes of cognitive control, such as deinhibition; (3) the bivalency effect was observed in the N1 and P2p components in the posterior region for the number parity task. These findings suggest that the modulation of cognitive control is task‐specific in task switching.

## Conflicts of Interest

The authors declare no conflicts of interest.

## Appendix

A 2 (block type: univalent, bivalent) × 3 (task type: shape color, number parity, letter case) repeated‐measures ANOVA was performed on the RT of univalent stimuli in the univalent blocks and the first three bivalent blocks (2–4) (see Table A1). The results showed that the main effect of task type was significant, *F*(2,68) = 49.67, *p* < 0.001, *η* ^2^ = 0.59. Multiple comparisons indicated that participants were faster in responding to stimuli in the shape color task and the letter task than in the number parity task (*ps* < 0.001), while there were no significant differences in the shape color task and the letter task (*p* > 0.1). The main effect of block type was significant, *F*(1,34) = 11.37, *p* = 0.002, *η* ^2^ = 0.25. The RT of the bivalent block (*M* = 546 ms) was significantly longer than that of the univalent block (*M* = 535 ms). There was no interaction between block type and task type (*p* = 0.230).

**TABLE A1:** Mean RTs (ms) for univalent stimuli in the univalent and bivalent blocks.

	Univalent block	Bivalent block (2–4)
Shape color task	513 (52)	527 (59)
Number parity task	565 (58)	580 (60)
Letter case task	526 (54)	533 (49)

## References

[pchj70011-bib-0001] Allport, A. , and G. Wylie . 2000. “Task Switching, Stimulus‐Response Bindings, and Negative Priming.” Attention and Performance 18: 33–70. 10.7551/mitpress/1481.003.0008.

[pchj70011-bib-0002] Botvinick, M. M. , T. S. Braver , C. S. Carter , D. M. Barch , and J. D. Cohen . 2001. “Evaluating the Demand for Control: Anterior Cingulate Cortex and Crosstalk Monitoring.” Psychological Review 108: 624–652. 11488380 10.1037/0033-295x.108.3.624

[pchj70011-bib-0003] Cai, W. , S. L. Warren , K. Duberg , A. Yu , S. P. Hinshaw , and V. Menon . 2023. “Both Reactive and Proactive Control Are Deficient in Children With ADHD and Predictive of Clinical Symptoms.” Translational Psychiatry 13, no. 1: 179. 10.1038/s41398-023-02471-w. 37236924 PMC10220086

[pchj70011-bib-0004] Cao, C. , W. Wen , A. Chen , et al. 2023. “Neuropsychological Alterations of Prolactinomas' Cognitive Flexibility in Task Switching.” Brain Sciences 13, no. 1: 82. 10.3390/brainsci13010082. 36672063 PMC9856801

[pchj70011-bib-0005] Chen, J. , S. Wu , and F. Li . 2022. “Cognitive Neural Mechanism of Backward Inhibition and Deinhibition: A Review.” Frontiers in Behavioral Neuroscience 16: 846369. 10.3389/fnbeh.2022.846369. 35668866 PMC9165717

[pchj70011-bib-0006] Clayson, P. E. , and M. J. Larson . 2013. “Psychometric Properties of Conflict Monitoring and Conflict Adaptation Indices: Response Time and Conflict N2 Event‐Related Potentials.” Psychophysiology 50: 1209–1219. 10.1111/psyp.12138. 23992600

[pchj70011-bib-0007] Dann, K. M. , A. Veldre , S. Miles , P. Sumner , P. Hay , and S. Touyz . 2023. “Measuring Cognitive Flexibility in Anorexia Nervosa: Wisconsin Card Sorting Test Versus Cued Task‐Switching.” Eating and Weight Disorders: EWD 28, no. 1: 60. 10.1007/s40519-023-01589-6. 37463996 PMC10354129

[pchj70011-bib-0008] Du, W. W. , and F. H. Li . 2018. “Bivalency Effect and Its Cognitive Mechanism.” Advances in Psychological Science 26, no. 11: 1969. 10.3724/SP.J.1042.2018.01969.

[pchj70011-bib-0009] Ebitz, R. B. , E. H. Smith , G. Horga , et al. 2020. “Human Dorsal Anterior Cingulate Neurons Signal Conflict by Amplifying Task‐Relevant Information.” BioRxiv. 10.1101/2020.03.14.991745.

[pchj70011-bib-0010] Egner, T. , M. Delano , and J. Hirsch . 2007. “Separate Conflict‐Specific Cognitive Control Mechanisms in the Human Brain.” NeuroImage 35, no. 2: 940–948. 10.1016/j.neuroimage.2006.11.061. 17276088

[pchj70011-bib-0011] Enger, T. 2009. “Multiple Conflict‐Driven Control Mechanism in the Human Brain.” Trends in Cognitive Sciences 12, no. 10: 374–380. 10.1016/j.tics.2008.07.001. 18760657

[pchj70011-bib-0012] Gajewski, P. D. , N. K. Ferdinand , J. Kray , and M. Falkenstein . 2018. “Understanding Sources of Adult Age Differences in Task Switching: Evidence From Behavioral and ERP Studies.” Neuroscience & Biobehavioral Reviews 92: 255–275. 10.1016/j.neubiorev.2018.05.029. 29885425

[pchj70011-bib-0013] Giller, F. , and C. Beste . 2019. “Effects of Aging on Sequential Cognitive Flexibility Are Associated With Fronto‐Parietal Processing Deficits.” Brain Structure and Function 224: 2343–2355. 10.1007/s00429-019-01910-z. 31218393

[pchj70011-bib-0014] Giller, F. , M. Mückschel , T. Ziemssen , and C. Beste . 2020. “A Possible Role of the Norepinephrine System During Sequential Cognitive Flexibility–Evidence From EEG and Pupil Diameter Data.” Cortex 128: 22–34. 10.1016/j.cortex.2020.03.008. 32311545

[pchj70011-bib-0015] Giordano, G. M. , P. Pezzella , L. Giuliani , et al. 2023. “Resting‐State Brain Activity Dysfunctions in Schizophrenia and Their Associations With Negative Symptom Domains: An fMRI Study.” Brain Sciences 13, no. 1: 83. 10.3390/brainsci13010083. 36672064 PMC9856573

[pchj70011-bib-0016] Gratton, G. , P. Cooper , M. Fabiani , C. S. Carter , and F. Karayanidis . 2018. “Dynamics of Cognitive Control: Theoretical Bases, Paradigms, and a View for the Future.” Psychophysiology 55, no. 3: e13016. 10.1111/psyp.13016. 29044552

[pchj70011-bib-0017] Grundy, J. G. , R. M. Barker , J. A. E. Anderson , and J. M. Shedden . 2019. “The Relation Between Brain Signal Complexity and Task Difficulty on an Executive Function Task.” NeuroImage 198: 104–113. 10.1016/j.neuroimage.2019.05.045. 31112787

[pchj70011-bib-0018] Grundy, J. G. , M. F. F. Benarroch , T. S. Woodward , P. D. Metzak , J. C. Whitman , and J. M. Shedden . 2013. “The Bivalency Effect in Task Switching: Event‐Related Potentials.” Human Brain Mapping 34, no. 5: 999–1012. 10.1002/hbm.21488. 22162123 PMC6869890

[pchj70011-bib-0019] Grundy, J. G. , and J. M. Shedden . 2014a. “A Role for Recency of Response Conflict in Producing the Bivalency Effect.” Psychological Research 78, no. 5: 679–691. 10.1007/s00426-013-0520-x. 24146081

[pchj70011-bib-0020] Grundy, J. G. , and J. M. Shedden . 2014b. “Support for a History‐Dependent Predictive Model of dACC Activity in Producing the Bivalency Effect: An Event‐Related Potential Study.” Neuropsychologia 57: 166–178. 10.1016/j.neuropsychologia.2014.03.008. 24686093

[pchj70011-bib-0021] Han, J. , Y. Dai , L. Xie , and F. Li . 2018. “Brain Responses Associated With Different Hierarchical Effects on Cues and Targets During Rule Shifting.” Biological Psychology 134: 52–63. 10.1016/j.biopsycho.2018.02.010. 29476839

[pchj70011-bib-0022] Jost, K. , V. Hennecke , and I. Koch . 2017. “Task Dominance Determines Backward Inhibition in Task Switching.” Frontiers in Psychology 8: 755–764. 10.3389/fpsyg.2017.00755. 28539907 PMC5423976

[pchj70011-bib-0023] Junker, F. B. , T. Schmidt‐Wilcke , A. Schnitzler , and J. Lange . 2023. “Temporal Dynamics of Oscillatory Activity During Nonlexical Language Decoding: Evidence From Morse Code and Magnetoencephalography.” Human Brain Mapping 44, no. 17: 6185–6197. 10.1002/hbm.26505. 37792277 PMC10619365

[pchj70011-bib-0024] Koch, I. , M. Gade , and A. M. Philipp . 2004. “Inhibition of Response Mode in Task Switching.” Experimental Psychology 51, no. 1: 52–58. 10.1027/1618-3169.51.1.52. 14959506

[pchj70011-bib-0025] Larson, M. J. , P. E. Clayson , and A. Clawson . 2014. “Making Sense of All the Conflict: A Theoretical Review and Critique of Conflict‐Related ERPs.” International Journal of Psychophysiology 93, no. 3: 283–297. 10.1016/j.ijpsycho.2014.06.007. 24950132

[pchj70011-bib-0026] Liu, P. , W. Yang , L. Chang , and A. Chen . 2013. “The Conflict Adaptation Effect Is Based on Specific‐Domain.” Journal of Psychological Science 36, no. 3: 696–701.

[pchj70011-bib-0027] Lobaugh, N. J. , R. West , and A. R. McIntosh . 2001. “Spatiotemporal Analysis of Experimental Differences in Event‐Related Potential Data With Partial Least Squares.” Psychophysiology 38, no. 3: 517–530. 10.1017/S0048577201991681. 11352141

[pchj70011-bib-0028] Mao, W. , and Y. P. Wang . 2011. “The Processing of Visual Double‐Feature Conflict Under Different Attentive Conditions: An Event‐Related Potential Study.” Chinese Journal of Mental Health 25, no. 2: 77–82.

[pchj70011-bib-0029] Mari‐Beffa, P. , and A. Kirkham . 2014. “The Mixing Cost as a Measure of Cognitive Control.” In Task Switching and Cognitive Control, edited by J. A. Grange and G. Houghton , 74–100. Oxford University Press. 10.1093/acprof:osobl/9780199921959.003.0004.

[pchj70011-bib-0030] McIntosh, A. R. , and N. J. Lobaugh . 2004. “Partial Least Squares Analysis of Neuroimaging Data: Applications and Advances.” NeuroImage 23: S250–S263. 10.1016/j.neuroimage.2004.07.020. 15501095

[pchj70011-bib-0031] Meier, B. , A. Rey‐Mermet , T. S. Woodward , R. Müri , and K. Gutbrod . 2013. “Episodic Context Binding in Task Switching: Evidence From Amnesia.” Neuropsychologia 51, no. 5: 886–892. 10.1016/j.neuropsychologia.2013.01.025. 23395937

[pchj70011-bib-0032] Meier, B. , T. S. Woodward , A. Rey‐Mermet , and P. Graf . 2009. “The Bivalency Effect in Task Switching: General and Enduring.” Canadian Journal of Experimental Psychology‐Revue Canadienne de Psychologie Expérimentale 63, no. 3: 201–210. 10.1037/a0014311. 19739903

[pchj70011-bib-0033] Metzak, P. D. , B. Meier , P. Graf , and T. S. Woodward . 2013. “More Than a Surprise: The Bivalency Effect in Task Switching.” Journal of Cognitive Psychology 25, no. 7: 833–842. 10.1080/20445911.2013.832196.

[pchj70011-bib-0034] Moretti, L. , I. Koch , M. Steinhauser , and S. Schuch . 2023. “Disentangling Task‐Selection Failures From Task‐Execution Failures in Task Switching: An Assessment of Different Paradigms.” Psychological Research 87, no. 3: 929–950. 10.1007/s00426-022-01708-5. 35835932 PMC10017612

[pchj70011-bib-0035] Moretti, L. , I. Koch , M. Steinhauser , and S. Schuch . 2024. “Stimulus‐Triggered Task Conflict Affects Task‐Selection Errors in Task Switching: A Bayesian Multinomial Processing Tree Modeling Approach.” Journal of Experimental Psychology: Learning, Memory, and Cognition 50, no. 2: 230–243. 10.1037/xlm0001245. 37155281

[pchj70011-bib-0036] Nicholson, R. , F. Karayanidis , E. Bumak , D. Poboka , and P. T. Michie . 2006. “ERPs Dissociate the Effects of Switching Task Sets and Task Cues.” Brain Research 1095, no. 1: 107–123. 10.1016/j.brainres.2006.04.016. 16714004

[pchj70011-bib-0037] Posner, M. I. , and S. E. Petersen . 1990. “The Attention System of the Human Brain.” Annual Review of Neuroscience 13: 25–42. 10.1146/annurev.ne.13.030190.000325. 2183676

[pchj70011-bib-0038] Posner, M. I. , and M. K. Rothbart . 2007. “Research on Attention Networks as a Model for the Integration of Psychological Science.” Annual Review of Psychology 58: 1–23. 10.1146/annurev.psych.58.110405.085516. 17029565

[pchj70011-bib-0039] Poulsen, C. , P. Luu , C. Davey , and D. M. Tucker . 2005. “Dynamics of Task Sets: Evidence From Dense‐Array Event‐Related Potentials.” Cognitive Brain Research 24, no. 1: 133–154. 15922166 10.1016/j.cogbrainres.2005.01.008

[pchj70011-bib-0040] Rey‐Mermet, A. , T. Koenig , and B. Meier . 2013. “The Bivalency Effect Represents an Interference‐Triggered Adjustment of Cognitive Control: An ERP Study.” Cognitive, Affective, & Behavioral Neuroscience 13: 575–583. 10.1016/j.cogbrainres.2005.01.008. 23584989

[pchj70011-bib-0041] Rey‐Mermet, A. , and B. Meier . 2012a. “The Bivalency Effect: Adjustment of Cognitive Control Without Response Set Priming.” Psychological Research 76, no. 1: 50–59. 10.1007/s00426-011-0322-y. 21347864

[pchj70011-bib-0042] Rey‐Mermet, A. , and B. Meier . 2012b. “The Bivalency Effect: Evidence for Flexible Adjustment of Cognitive Control.” Journal of Experimental Psychology: Human Perception and Performance 38, no. 1: 213–221. 10.1037/a0026024. 22060142

[pchj70011-bib-0043] Rey‐Mermet, A. , and B. Meier . 2014a. “Age Affects the Adjustment of Cognitive Control After a Conflict: Evidence From the Bivalency Effect.” Aging, Neuropsychology, and Cognition 22, no. 1: 72–94. 10.1080/13825585.2014.889070. 24559329

[pchj70011-bib-0044] Rey‐Mermet, A. , and B. Meier . 2014b. “More Conflict Does Not Trigger More Adjustment of Cognitive Control for Subsequent Events: A Study of the Bivalency Effect.” Acta Psychologica 145: 111–117. 10.1016/j.actpsy.2013.11.005. 24333810

[pchj70011-bib-0045] Rey‐Mermet, A. , and B. Meier . 2017. “How Long‐Lasting Is the Post‐Conflict Slowing After Incongruent Trials? Evidence From the Stroop, Simon, and Flanker Tasks.” Attention, Perception, & Psychophysics 79, no. 7: 1945–1967. 10.3758/s13414-017-1348-z. 28608273

[pchj70011-bib-0046] Scannella, S. , J. Pariente , X. De Boissezon , et al. 2016. “N270 Sensitivity to Conflict Strength and Working Memory: A Combined ERP and sLORETA Study.” Behavioural Brain Research 297: 231–240. 10.1016/j.bbr.2015.10.014. 26477377

[pchj70011-bib-0047] Schuch, S. , D. Dignath , M. Steinhauser , and M. Janczyk . 2019. “Monitoring and Control in Multitasking.” Psychonomic Bulletin & Review 26, no. 1: 222–240. 10.3758/s13423-018-1512-z. 30066081

[pchj70011-bib-0048] Sheth, S. A. , M. K. Mian , S. R. Patel , et al. 2012. “Human Dorsal Anterior Cingulate Cortex Neurons Mediate Ongoing Behavioral Adaptation.” Nature 488, no. 7410: 218–221. 10.1038/nature11239. 22722841 PMC3416924

[pchj70011-bib-0049] Smith, E. H. , G. Horga , M. J. Yates , et al. 2019. “Widespread Temporal Coding of Cognitive Control in the Human Prefrontal Cortex.” Nature Neuroscience 22, no. 11: 1883–1891. 10.1038/s41593-019-0494-0. 31570859 PMC8855692

[pchj70011-bib-0050] Strobach, T. , M. Wendt , and M. Janczyk . 2018. “Multitasking: Executive Functioning in Dual‐Task and Task Switching Situations.” Frontiers in Psychology 9: 108. 10.3389/fpsyg.2018.00108. 29497390 PMC5818435

[pchj70011-bib-0051] Swainson, R. , R. Cunnington , G. M. Jackson , et al. 2003. “Cognitive Control Mechanisms Revealed by ERP and fMRI: Evidence From Repeated Task‐Switching.” Journal of Cognitive Neuroscience 15, no. 6: 785–799. 10.1162/089892903322370717. 14511532

[pchj70011-bib-0052] Syrov, N. , L. Yakovlev , V. Nikolaeva , A. Kaplan , and M. Lebedev . 2022. “Mental Strategies in a P300‐BCI: Visuomotor Transformation Is an Option.” Diagnostics 12, no. 11: 2607. 10.3390/diagnostics12112607. 36359454 PMC9689852

[pchj70011-bib-0053] Vandierendonck, A. , B. Liefooghe , and F. Verbruggen . 2010. “Task Switching: Interplay of Reconfiguration and Interference Control.” Psychological Bulletin 136, no. 4: 601–626. 10.1037/a0019791. 20565170

[pchj70011-bib-0054] Wang, H. J. , Y. P. Wang , J. Kong , L. L. Cui , and S. J. Tian . 2001. “Enhancement of Conflict Processing Activity in Human Brain Under Task Relevant Condition.” Neuroscience Letters 298, no. 3: 155–158. 10.1016/s0304-3940(00)01757-2. 11165430

[pchj70011-bib-0055] Wang, Y. P. , S. J. Tian , H. J. Wang , L. L. Cui , Y. Y. Zhang , and X. Zhang . 2003. “Event‐Related Potentials Evoked by Multi‐Feature Conflict Under Different Attentive Conditions.” Experimental Brain Research 148, no. 4: 451–457. 10.1007/s00221-002-1319-y. 12582828

[pchj70011-bib-0056] Wolff, N. , F. Giller , J. Buse , V. Roessner , and C. Beste . 2018. “When Repetitive Mental Sets Increase Cognitive Flexibility in Adolescent Obsessive–Compulsive Disorder.” Journal of Child Psychology and Psychiatry 59, no. 9: 1024–1032. 10.1111/jcpp.12901. 29603217

[pchj70011-bib-0057] Woodward, T. S. , B. Meier , C. Tipper , and P. Graf . 2003. “Bivalency Is Costly: Bivalent Stimuli Elicit Cautious Responding.” Experimental Psychology 50, no. 4: 233–238. 10.1026//1618-3169.50.4.233. 14587170

[pchj70011-bib-0058] Woodward, T. S. , P. D. Metzak , B. Meier , and C. B. Holroyd . 2008. “Anterior Cingulate Cortex Signals the Requirement to Break Inertia When Switching Tasks: A Study of the Bivalency Effect.” NeuroImage 40, no. 3: 1311–1318. 10.1016/j.neuroimage.2007.12.049. 18291678

[pchj70011-bib-0059] Xie, L. , B. Cao , Z. Li , and F. Li . 2020. “Neural Dynamics of Cognitive Control in Various Types of Incongruence.” Frontiers in Human Neuroscience 14: 214. 10.3389/fnhum.2020.00214. 32581754 PMC7291779

[pchj70011-bib-0060] Yancey, J. R. , C. B. Bowyer , K. E. Roberts , et al. 2022. “Boldness Moderates Cognitive Performance Under Acute Threat: Evidence From a Task‐Switching Paradigm Involving Cueing for Shock.” Journal of Experimental Psychology: Human Perception and Performance 48, no. 6: 549–562. 10.1037/xhp0000995. 35446089

[pchj70011-bib-0061] Zhuo, B. X. , Y. Chen , M. Q. Zhu , B. H. Cao , and F. H. Li . 2021. “Response Variations Can Promote the Efficiency of Task Switching: Electrophysiological Evidence.” Neuropsychologia 156: 107828. 10.1016/j.neuropsychologia.2021.107828. 33727087

